# Protocol, rationale and design of SELPHI: a randomised controlled trial assessing whether offering free HIV self-testing kits via the internet increases the rate of HIV diagnosis

**DOI:** 10.1186/s12879-018-3433-x

**Published:** 2018-10-23

**Authors:** Michelle M. Gabriel, David T. Dunn, Andrew Speakman, Leanne McCabe, Denise Ward, T. Charles Witzel, Justin Harbottle, Simon Collins, Mitzy Gafos, Fiona M. Burns, Fiona C. Lampe, Peter Weatherburn, Andrew Phillips, Sheena McCormack, Alison J. Rodger

**Affiliations:** 1 MRC Clinical Trials Unit at UCL, London, UK; 2 Centre for Clinical Research, Epidemiology, Modelling and Evaluation, Institute for Global Health, UCL, London, UK; 3 Department of Social and Environmental Health Research, Sigma Research, Faculty of Public Health & Policy, London School of Hygiene and Tropical Medicine, London, UK; 4 Terrence Higgins Trust, London, UK; 5 HIV i-Base, London, UK; 6 Department of Global Health and Development, London School of Hygiene and Tropical Medicine, Faculty of Public Health and Policy, London, UK; 7 Royal Free London NHS Foundation Trust, London, UK; 8 Trial Sponsor – University College London via MRC Clinical Trials Unit at UCL, Institute of Clinical Trials & Methodology, 90 High Holborn, 2nd Floor, London, WC1V 6LJ UK

**Keywords:** HIV, Self-testing, HIVST, MSM, Diagnosis, Prevalent, Incident

## Abstract

**Background:**

Among men who have sex with men (MSM) in the UK, an estimated 28% have never tested for HIV and only 27% of those at higher risk test at least every 6 months. HIV self-testing (HIVST), where the person takes their own blood/saliva sample and processes it themselves, offers the opportunity to remove many structural and social barriers to testing. Although several randomised controlled trials are assessing the impact of providing HIVST on rates of HIV testing, none are addressing whether this results in increased rates of HIV diagnoses that link to clinical care. Linking to care is the critical outcome because it is the only way to access antiretroviral treatment (ART). We describe here the design of a large, internet-based randomised controlled trial of HIVST, called SELPHI, which aims to inform this key question.

**Methods/design:**

The SELPHI study, which is ongoing is promoted via social networking website and app advertising, and aims to enroll HIV negative men, trans men and trans women, aged over 16 years, who are living in England and Wales. Apart from the physical delivery of the test kits, all trial processes, including recruitment, take place online. In a two-stage randomisation, participants are first randomised (3:2) to receive a free baseline HIVST or no free baseline HIVST. At 3 months, participants allocated to receive a baseline HIVST (and meeting further eligibility criteria) are subsequently randomised (1:1) to receive the offer of regular (every 3 months) free HIVST, with testing reminders, versus no such offer. The primary outcome from both randomisations is a laboratory-confirmed HIV diagnosis, ascertained via linkage to a national HIV surveillance database.

**Discussion:**

SELPHI will provide the first reliable evidence on whether offering free HIVST via the internet increases rates of confirmed HIV diagnoses and linkage to clinical care. The two randomisations reflect the dual objectives of detecting prevalent infections (possibly long-standing) and the more rapid diagnosis of incident HIV infections. It is anticipated that the results of SELPHI will inform future access to HIV self-testing provision in the UK.

**Trial registration:**

DOI 10.1186/ISRCTN20312003 registered 24/10/2016.

## Background

The United Nations (UN) 90–90-90 targets aim by 2020, that 90% of all people living with HIV (PLWH) are diagnosed, that 90% of people diagnosed with HIV are on ART, and that 90% of those on ART have a suppressed viral load [1]. The first target (90% diagnosis) remains the key challenge with global estimates of 47% of PLWH being unaware of their infection. Knowledge of one’s own HIV status and accessing ART benefits health on both an individual and population level. People who are unaware of their status are estimated to contribute disproportionally to new transmissions (between 60 and 80%) [2]. In the 2017 PHE (Public Health England) report on HIV, it was estimated that 10% of gay/bisexual men who have sex with men (MSM) were unaware of their HIV status. Although this percentage has decreased since 2010, testing is often less frequent than current recommendations. For example, UK guidelines [3] currently recommend annual HIV testing for MSM, and three-monthly testing for those considered ‘at higher risk’ (a definition that includes condomless (CL) anal sex with a new partner, diagnosis of new STI or chemsex drug use). In UK MSM, an estimated 28% have never tested for HIV and only a quarter of men at ‘higher risk’ of HIV infection test even 6-monthly (27%) [4–6]. Late diagnosis of HIV also remains a problem in the UK; 32% of all MSM diagnosed with HIV in 2016 (663/2,096) had CD4 counts below 350 mm^3^, which is associated with greater morbidity and mortality than those who are diagnosed earlier in the course of infection. All guidelines now recommend that PLWH commence ART at diagnosis as this has been shown to be beneficial to individual health even at high CD4 counts [7].

HIV diagnoses in MSM in London have fallen since 2016 and this is thought to be a combination of increased rates of HIV testing, rapid initiation of ART when HIV is diagnosed (which reduces transmission risk through sex to almost zero) and increasing use of pre-exposure prophylaxis (PrEP) [8]. Rates of HIV diagnosis in MSM outside London have also reduced but to a lesser degree [9]. Expanding ways for MSM to test for HIV outside of traditional settings (such as GUM clinics) has been a focus for over a decade and there is now a national self-sampling service. This involves an individual taking their own test sample which they post back to the relevant laboratory for testing and are subsequently contacted with the result.

A further approach is to offer HIV self-testing (HIVST) where the person not only takes their own blood/saliva sample but also processes it themselves using a self-testing kit, and obtains the results immediately. A potential advantage of HIVST is that, removing structural and social barriers to testing and increasing associated privacy and convenience, may lead to increased testing [10, 11]. HIVST is also an opportunity for prevention synergies with the availability of PrEP (which requires frequent testing), and for harm reduction strategies, such as sexual partner screening. The WHO now incorporate HIVST into its global HIV testing guidelines as a ‘supplementary’ or ‘additional’ option [12] and has described it as “an empowering and innovative way to help achieve the first of the United Nations 90–90–90 treatment targets” [13].

HIVST also has a number of potential challenges, although empirical data are lacking. Firstly, a person who has a reactive HIVST requires confirmatory HIV testing to link to care which relies on the individual seeking more traditional testing as a gateway to care and support. It remains unknown what proportion of individuals who obtain a reactive result on HIVST link to care in a timely manner [14]. A further issue may be the potential for social and emotional harms from a reactive test in the absence of counselling, or coercion to test from a partner. HIVST may also be a missed opportunity for STI screening and advice about risk management due to fewer visits to GUM clinic settings, which may put MSM at increased risk of other STIs. HIVST kit accuracy is also an area of concern, as the window period is prolonged in comparison to 4th generation tests (antibody and P24 antigen test) and the sensitivity is relatively low, particularly with oral fluid HIVST in early infection or in breakthrough infections on tenofovir-based PrEP as antibody levels may be low. This is particularly important during early infection, when the risk of onward transmission is markedly increased.

### Existing evidence base

Evidence suggests that HIVST is acceptable to MSM and other key populations at risk of HIV globally both in high and low-income settings [10, 15, 16]. However, despite the theoretical benefits of HIVST, there are limited European data exploring potential HIVST acceptability, as well as the values and preferences of MSM or trans people at risk of HIV infection on the potential impacts of self-testing approaches in the UK [17–19]. There is also a lack of evidence on whether HIVST increases rates of HIV diagnosis in populations at risk of HIV. It is also unknown whether it is cost-effective for the NHS to provide free or subsidised HIVST kits. Observational studies, using follow-up surveys, have documented the number of self-reported reactive tests as a proportion of the number of HIVST kits sent out [20]. However, there is likely to be selection bias in those responding to surveys and it is not known how many individuals would have sought and obtained their HIV diagnosis through another testing modality.

There are four on-going or recently reported RCTs of HIVST in MSM in high resource settings (three in the US and one in Australia) [21–24]. All use self-reported frequency of testing as the primary outcome comparing HIVST to standard of care. These studies therefore do not address the key question of whether provision of HIVST can increase rates of HIV diagnoses that link to clinical care, which is the gateway to ART. We describe here the design of a large, internet-based randomised controlled trial of HIVST, which aims to inform this question.

### Rationale

The primary aim of SELPHI is to measure the impact of HIVST on new confirmed HIV diagnoses linked to clinical care by addressing the following key questions:

- Is the online promotion and postal delivery of free HIV self-test kits (with testing reminders) feasible and acceptable?
- Will the offer of a single free HIV self-test at enrolment lead to the confirmed diagnosis of prevalent HIV infections and entry to standard HIV clinical care?
- Among seronegative individuals at high risk of acquiring HIV infection, will the offer of regular free self-tests with testing reminders result in more rapid confirmed diagnosis of an incident HIV infection and entry in to standard HIV clinical care?
- What data can be generated to inform key parameters for a cost effectiveness model?

Subsidiary objectives include: describing the usage and acceptability of HIVST; determining if the offer of free HIVST kits affects the overall frequency of HIV testing and testing options utilised; assessment of post-test linkage with counselling and treatment services; assessing whether free HIVST kits affects the frequency of STI screening or the frequency of condomless sex; investigating the impact of demographic, socio-economic, health-related factors, and sexual risk behaviours on testing behaviours.

## Methods/design

### Design

**Fig. 1:**
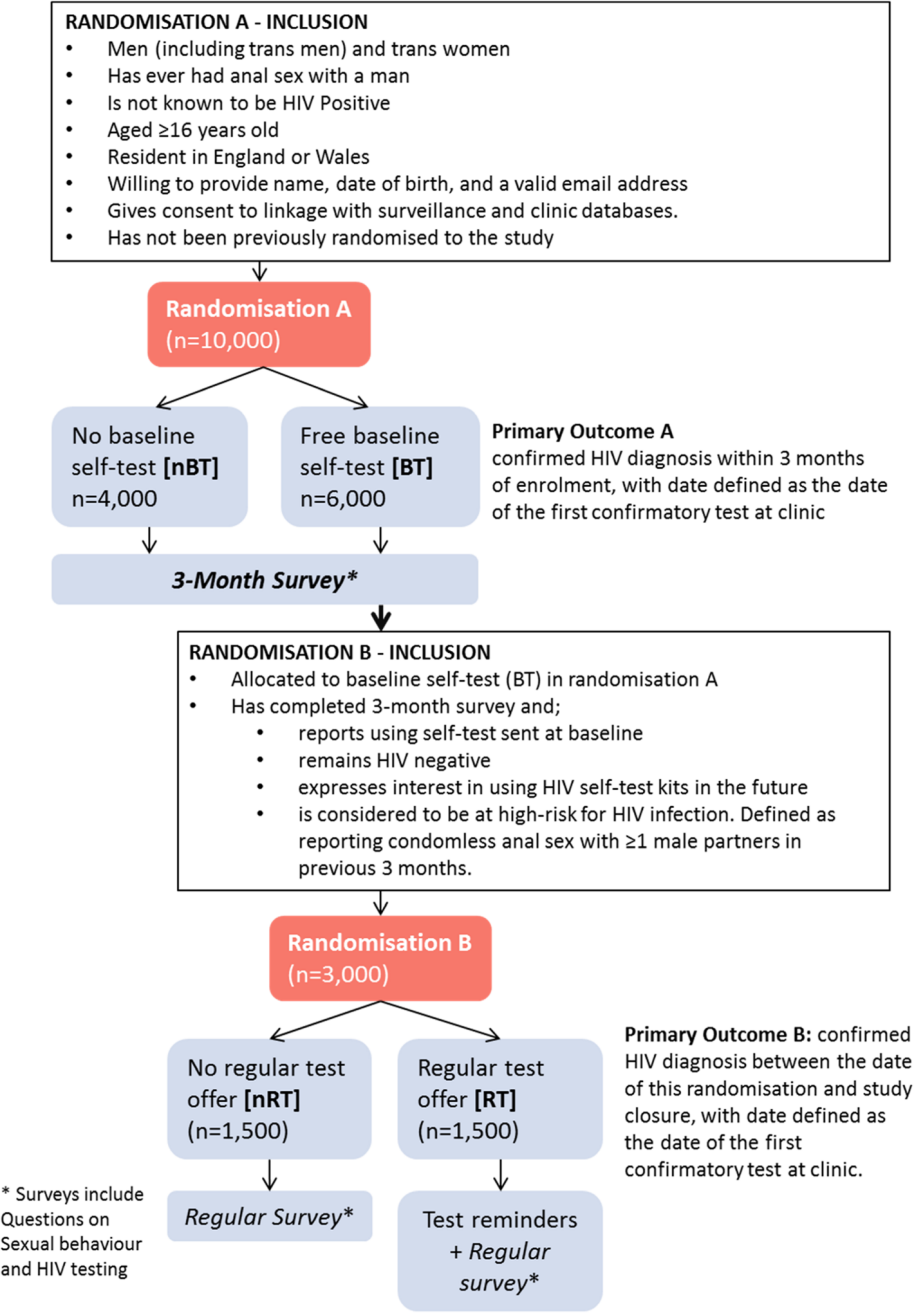
Trial Schema

SELPHI is an ongoing open-label parallel group randomised controlled trial with a two-stage simple randomisation aiming to enrol 10,000 participants (Fig. 1). Randomisation A takes place at enrolment, with participants randomly allocated (in a 3:2 ratio) to the offer of a free baseline HIV self-test (BT) versus no offer of a free baseline HIV self-test (nBT). An unequal allocation ratio was chosen so that a majority of those agreeing to participate would receive a free self-test and to increase the number of participants eligible for Randomisation B. This second randomisation occurs at month 3 after enrolment, and is more restrictive, being open only to participants who were initially allocated to the BT group in Randomisation A who complete the 3-month survey, and who meet additional eligibility criteria, including being at high risk of incident HIV infection, assessed at 3 months. Eligible participants are randomised (1:1) to receive the offer of regular (immediately and every 3 months thereafter) free HIV self-tests + testing reminders (RT) versus no such offer (nRT).

### Primary outcome measure

The primary outcome for both randomisations is a laboratory-confirmed HIV diagnosis, with date of diagnosis defined as the date of the first confirmatory test at clinic. This key feature of SELPHI distinguishes it from other randomised trials of HIVST.

### Inclusion criteria

The inclusion criteria for SELPHI are broad in order to maximise generalisability and are detailed in Table 1. The residency restriction was a requirement of the funding body. The consent for linkage to Public Health Databases was essential as this is the main mechanism for determining the trial primary endpoints. The criterion in Randomisation B of reporting at least one male condomless anal sex act in the previous 3 months is intended to identify individuals at higher risk of acquiring HIV infection.

**Table 1:** Inclusion criteria

Randomisation A	Randomisation B
ᅟ• Male (including trans men) and trans women ᅟ• Aged ≥16 years old ᅟ• Resident in England or Wales ᅟ• Not known to be HIV-positive ᅟ• Has ever had anal sex with a man ᅟ• Willing to provide name, date of birth, and a valid email address ᅟ• Consent for linkage to surveillance and clinic databases held by Public Health England ᅟ• Not previously randomised to the study	• Allocated to baseline self-test (BT) in Randomisation A • Completes the first survey after 3-months and: ᅟο Reports using self-test sent at baseline ᅟο Remains HIV-negative ᅟο Reports condomless anal sex with ≥1 male partners in previous 3 months ᅟο Interested in using HIV self-test kits in the future

### Study procedures

#### Recruitment and enrolment

**Fig. 2:**
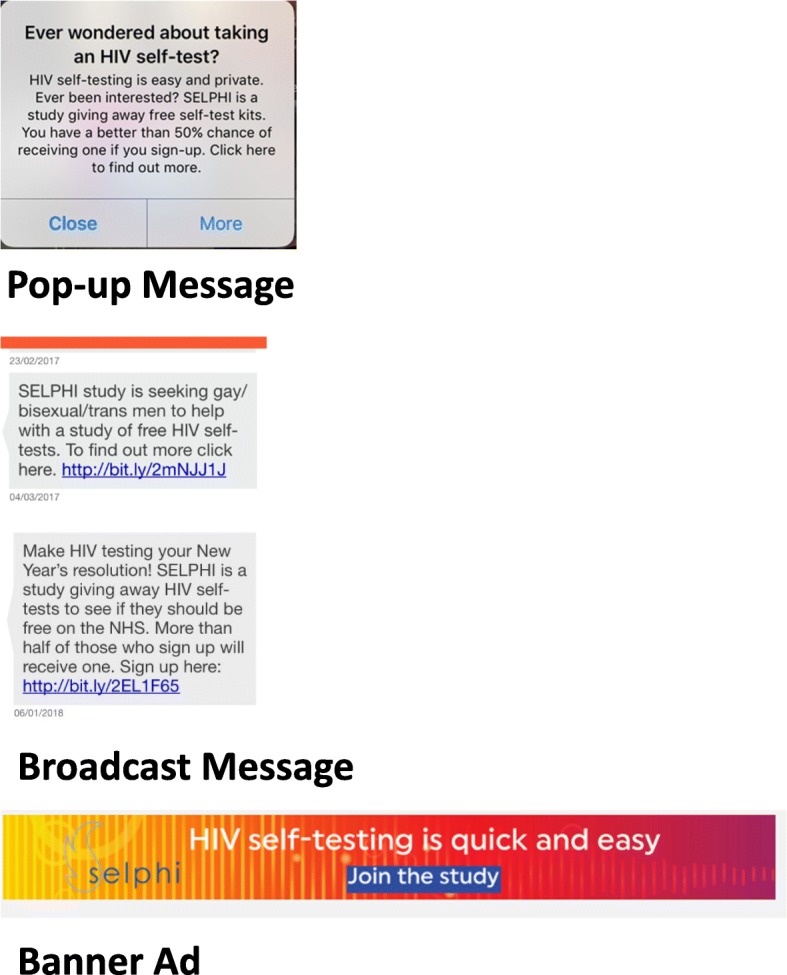
Advertising Samples

SELPHI is an internet-based study using advertising campaigns, placed on social networking websites and via mobile phone applications designed to facilitate sexual and social contact, to recruit participants for example Facebook, Grindr, Hornet and community webpages. Advertising is tailored to attract individuals from a broad spectrum of MSM and trans people. Depending on the advertising platform, messages take a number of forms: “Broadcast Message” (sent directly to an individual’s “inbox” in a particular app), “Pop-up Message” (pop-up message which shows when an individual logs in to an app), “Banner Ad” (shown on screen within an app or website whilst a user is online). Examples of adverts are shown in Fig. 2.

Participants are directed to the study registration page where they are asked to complete a two-stage sign-up process. All data are collected in electronic surveys hosted by Demographix Ltd. The first stage provides information about the study, assesses eligibility, obtains informed consent, and requests an email address. An email is immediately sent to this address with a link to complete the second stage of the process. This serves to validate the email address provided by the participant, which is the means of all future communication throughout the trial. In the second stage, further demographic and behavioural characteristics are collected (summarised in Table 2). Once the second stage is completed, participants are randomised and allocated to receive a free baseline HIV self-test (BT) or no free baseline test (nBT). Those allocated to BT are directed to provide postal details for shipment of the test kit; those allocated to nBT are directed to an area on the study website (www.selphi.org) which provides information on how to obtain an HIV test in other ways (e.g. local GUM clinics).

**Table 2:** Summary of variables collected on electronic surveys

Variable	Patient Group	Time point(s)
Age	ALL	Baseline
Soundex	ALL	Baseline
Postcode	ALL	Baseline
Country of Birth	ALL	Baseline
Length of residency in the UK	ALL	Baseline
Ethnicity	ALL	Baseline
Highest Educational Qualification	ALL	Baseline
Sexual Identity	ALL	Baseline
Timing of last HIV test	ALL	Baseline
Timing of last STI screen	ALL	Baseline
Number condomless anal intercourse partners (last 3 months)	ALL	Baseline
PrEP and PEP usage	ALL	Baseline
Confirmation of HIVST Kit receipt	BT/RT	2-weeks post kit shipment
	BT	3-month post baseline
	RT	3-monthly post Randomisation B entry
Confirmation of HIVST Kit usage	BT/RT	2-weeks post kit shipment
	BT	3-month post baseline
	RT	3-monthly post Randomisation B entry
Self-reported HIVST result	BT/RT	2-weeks post kit shipment
	BT	3-month post baseline
	RT	3-monthly post Randomisation B entry
Kits receipt & usage experiences	BT	3-month post baseline
	RT	3-monthly post Randomisation B entry
Timing of HIV tests in last 3 months (not including HIVST)	BT/NBT	3-month post baseline
	RT/NRT	3-monthly post Randomisation B entry
Self-reported HIV positive test result (from any other source)	BT/NBT	3-month post baseline
	RT/NRT	3-monthly post Randomisation B entry
Timing of STI tests in last 3 months	BT/NBT	3-month post baseline
	RT/NRT	3-monthly post Randomisation B entry
Number condomless anal intercourse partners (last 3 months)	BT/NBT	3-month post baseline
	RT/NRT	3-monthly post Randomisation B entry
Interest in future HIVST If available	BT/NBT	3-month post baseline
Offer of another free HIVST	RT	Randomisation B Entry
	RT	3-monthly post Randomisation B entry

#### HIV self-testing kits

In the UK, HIVST was legalised in April 2014, and the first CE marked kit (BioSURE® HIV Self Test, BioSURE, United Kingdom) was released to the UK market in April 2015. The BioSURE® HIV Self Test kit is classed as a 2nd generation test (an antibody immunoassay detecting HIV 1/2 antibodies from approximately 28 days after infection), uses a whole blood sample and retail at £30–£35. Further HIVST kits have subsequently obtained a CE mark, including the blood based INSTI HIV Self Test (bioLytical Laboratories, Canada) which detects anti-HIV-1 IgM antibodies as well as anti-HIV-1 IgG (a 3rd generation assay) and can detect HIV infection from 21 days after infection.

The HIV self-testing kit used in the study is the BioSURE® HIV Self Test which is CE marked and licensed for use in the UK. The test comprises a paper test strip inside a plastic barrel, and is performed by mixing a small drop of blood with test reagents contained in the buffer pot where the liquid reagents are absorbed by the paper strip. When the test is completed, two lines can appear on the paper test strip. The upper line (the Control line) becomes visible if the test has been performed correctly. The lower line (the Test line) becomes visible if the applied sample contains sufficient antibodies to HIV. The BioSURE® HIV self-test product insert estimates its sensitivity to be 99.7% (95% CI 98.9–100).

In addition to written information provided with the test kit, an online video providing instructions on kit use (produced by BioSURE) is also promoted to participants on joining the study and is available on the study website (https://youtu.be/N4CAqsmN_6g).

#### Follow up

All follow-up in SELPHI is conducted via online surveys which are only accessible using a unique personalised URL sent to participants by email. The content of surveys depends on the randomised allocation and uses conditional branching to create a customised path through the survey based on responses to earlier questions.

Following entry to Randomisation A participants randomised to BT are asked to provide a postal address for shipment of their free HIVST (which arrives within 5–7 days). Participants in the BT arm receive an invite to complete a short follow-up 2 weeks later, primarily to confirm that they received the HIVST and to ascertain if they have used it. Participants in the control arm are provided with signposts to allow them to access other options for HIV testing. All participants in Randomisation A are followed up at 3 months in an online survey, asking about HIV tests (type and number) conducted since baseline and the results of these tests, STI testing and the number of sexual partners since baseline. Questions are identical in the two groups, except the BT group is also asked to rate their experiences in receiving and using the HIVST. This survey also includes questions which determine eligibility for Randomisation B (refer to Table 2). Participants who do not enter Randomisation B receive no further surveys apart from one at the end of the study. Participants randomised to RT are immediately informed that they can order a further free HIVST now and every 3 months subsequently.

Participants in both arms (nRT and RT) of Randomisation B receive an invite to complete a survey every 3 months until the end of the study. For those in the RT arm, both the invitation and survey include a reminder to test and the offer of another free HIVST kit. The process for obtaining a kit is the same as the process at baseline; kits are not sent automatically but the participant “orders” another kit if they wish to. Participants are not obliged to order a kit every 3 months, if they choose not to receive another kit they will continue to be offered kits three-monthly. Following every kit order the participant will receive a 2-week follow-up as with their first kit. Follow-up in Randomisation B will continue until the last participant randomised is followed-up for 2 years. All participants who have not tested positive during the study will be sent an email at the end of the study inviting them to complete a final survey and to thank them for their participation.

If a participant reports testing HIV positive at any point during the study they are directed to resources where they can access support (e.g. THT Direct 24-h Helpline) and are no longer invited to complete any further follow-up surveys.

#### Determination of primary outcomes

Primary outcomes will be identified by linking the personal identifiers collected in SELPHI to the national (England and Wales) HIV surveillance database maintained by PHE, who are collaborators on the trial. Linkage will be performed by a computer algorithm, primarily based on date of birth and patient surname (encoded to Soundex). Putative links will be confirmed manually by matching on common variables, including geographical region, ethnicity, gender, and initials. On confirmed matched cases, PHE will return information on date of diagnosis, region of diagnosis, CD4 count and viral load at diagnosis, whether participants have linked to care and initiated treatment, and GUM clinic attendance history. Furthermore, at each follow-up survey participants are asked about any HIV tests taken and any HIV-positive diagnoses. Consistency between this information and that recorded in PHE databases will be cross-checked. A self-reported diagnosis will not be accepted as a primary outcome if it cannot be matched to a confirmatory test in the PHE database or from a local clinic.

### Patient and public involvement (PPI)

As per the NIHR INVOLVE guidelines, patient and public involvement (PPI) was sought during the trial development. PPI representatives from HIV i-Base, NAM and other organisations are included in programme management group and the trial management group. These community members led the establishment of a study specific Community Advisory Group (CAG) and developed other public involvement models. Patient and public involvement was integrated throughout the study and budgeted in the initial grant application. For example, it informed the development of the trial design, protocol, participant information and consent materials, surveys, recruitment strategy and advertising materials. This involvement notably expanded the entry criteria to include transgender women, even though the original grant was limited to gay and bisexual men. The change was driven by the lack of specific research and access for this population and the precedent of broader inclusion in other prevention studies (for example with PrEP).

### Ethical considerations

The potential adverse psychological consequences of a reactive test result were considered during the process of obtaining CE-marking for the BioSURE® self-test kit, but the regulatory authorities were satisfied that benefit of an individual knowing that they had HIV outweighed the small risk of harm. A second potential adverse consequence is that people who obtain a reactive test result might not subsequently attend clinical services for confirmatory HIV testing and therefore might not engage with care. Consequently, participants who report a reactive HIV test result in the study are directed to appropriate resources through the study website. These include guidance on how to find local GUM clinics and links to the Terrance Higgins Trust (THT) Direct hotline and the NHS Direct service to assist them in dealing with a new diagnosis and accessing confirmatory testing and HIV care services. There was extensive discussion around whether the lower age limit should be 18 years (standard age for consent for adult medical research) or 16 years (the legal age for consent for consensual sex). The latter age was chosen following consultation with the SELPHI CAB and then the Ethics Committee that approved the protocol.

Identifiable and sensitive data are collected within the trial, including questions on sexual behaviours, recreational drug use and HIV status. In compliance with all relevant legislation (including the Data Protection Act), data are stored securely within appropriate systems operated by Demographix Ltd. and University College London (Data Safe Haven) which meet the ISO27001 information security standard as a minimum. In datasets provided to PHE for the purposes of linkage, the minimum number of data fields are transferred, forenames are redacted to initials and surnames are encoded to Soundex, a phonetic algorithm which indexes names by sound as pronounced in English. All personal identifiers are stripped from datasets produced for statistical analyses.

Through patient and public involvement, it was decided that describing the full complexity of the trial design would result in information overload and could hinder recruitment. Potential participants are therefore simply informed that they will be randomised to one or more self-tests to which they are asked to consent, rather than explicit and separate consents for Randomisation A and Randomisation B.

### Statistical analysis

The primary analyses will compare the randomised groups as allocated (intention to treat, ITT) in terms of a confirmed HIV diagnosis. Specifically, the primary outcome for Randomisation A is a confirmed HIV diagnosis within 3 months of the date of randomisation i.e. before the 3-month survey, which could influence testing behaviour in the nBT group, is sent out. The difference between randomised arms will be tested by a chi-squared test for comparison of proportions. Logistic regression analysis will be used to explore the effect of other covariates and potential interactions with randomisation arm. As the offer of a free test at enrolment could theoretically also affect future testing behaviour, a secondary survival analysis will examine the time to confirmed diagnosis.

The primary outcome for Randomisation B (RT versus nRT) is time to confirmed diagnosis of HIV from the date of randomisation. The analysis includes information on participants who do not experience the event, using time-to-event methods. Ideally, we would describe the interval from the time of acquisition of HIV infection rather than randomisation, but this is not generally observed. We note that the number and timing of endpoints is a function both of underlying HIV incidence and the interval between infection and diagnosis. If the self-testing intervention affects the former this will induce a difference between the randomised groups, even if there is no impact on diagnosis rates; although we cannot exclude the possibility of such a mechanism we consider it to be unlikely. The difference between randomised arms will be tested by a log rank test, supplemented by Cox regression models to examine the effect of covariates. The (administrative) censoring date for participants who do not experience the primary outcome will depend on the calendar date when linkage to PHE datasets is performed (see Determination of primary outcomes).

### Sample size

The standard approach to sample size is to first pre-specify key parameters, including the desired statistical power, and then calculate the sample size. However, this approach was not practicable as we were constrained by the budget for the HIVST kits and as certain key parameters were highly uncertain. Instead, for the pre-determined sample size of 10,000 we have estimated the statistical power over plausible ranges of values for these parameters.

The power of the analysis of Randomisation A is a function of underlying HIV seroprevalence and the proportion of seropositive participants in the BT and nBT groups diagnosed within 3 months. We considered HIV seroprevalence values between 1.5 and 2.5%, based on HIV self-sampling in the UK, [25] and proportions diagnosed between 20 and 50% in the nBT group and between 50 and 80% in the BT group. Table 3 shows the statistical power for various combinations of these proportions when HIV seroprevalence is 2.0%. In general, power is acceptably high when the difference between the BT and nBT groups is at least 30% (in absolute terms).

**Table 3:** Power (%) to detect a difference between BT and nBT groups in Randomisation A

Diagnosis rate in nBT group (%)	Diagnosis rate in BT group (%)			
	50	60	70	80
20	91	99	100	100
30	53	83	96	99
40	16	45	75	92
50	5	14	39	68

Power to detect a difference at 2α = 0.05 by chi-squared test

Assumes seroprevalence rate = 2%

We have assumed that 3,000 participants will enter Randomisation B i.e. 50% of those enrolled in the BT group meet the additional eligibility criteria. We used simulation to estimate the statistical power for this randomisation as an analytical approach was not tractable. As repeat HIV self-tests can only affect the time to diagnosis of participants who become infected during the study, a key parameter is the underlying HIV incidence rate. Values between 1.5 and 3.0 per 100 person-years (PY) were explored, based on estimates among MSM attending GUM clinics in England. Another important parameter is the interval between infection and diagnosis in the nRT group: this was assumed to follow a Weibull distribution with a shape parameter of 0.4 (to produce a higher initial rate of detection of infection) and a median ranging from 1.0 to 2.5 years [26, 27]. The corresponding interval in the RT group was determined by the uptake of the offer of repeat tests and the proportion linking to care following a reactive self-test. Table 4 shows the statistical power and other analytical outputs as a function of key parameters.

**Table 4:** Power to detect a difference between RT and nRT groups in Randomisation B

HIV Incidence (per 100 PY)	Median time to diagnosis (years) in nRT group	Power (%)	Estimated HIV infections per group	Median number HIV diagnoses (nRT/RT)	Median hazard ratio (RT versus nRT)
1.5	1.0	56	53	24/40	1.73
	1.5	72	53	21/40	1.91
	2.0	82	53	19/40	2.08
	2.5	87	53	18/40	2.21
2.0	1.0	75	71	30/53	1.70
	1.5	83	71	28/52	1.94
	2.0	91	71	26/52	2.08
	2.5	93	71	24/51	2.20
2.5	1.0	83	89	38/66	1.73
	1.5	93	89	34/65	1.92
	2.0	96	89	32/65	2.09
	2.5	98	89	30/64	2.25
3.0	1.0	89	107	46/78	1.74
	1.5	96	107	41/77	1.92
	2.0	98	107	38/77	2.09
	2.5	99	107	36/77	2.23

Power to detect a difference at 2α = 0.05 by log-rank test

## Discussion

Globally, SELPHI is the largest RCT evaluating the offer of free HIVST kits via the internet, and has uniquely been designed to assess the impact of this intervention on HIV diagnosis with linkage to clinical care and thus access to early ART.

SELPHI was challenging to design and the results will also need to be interpreted carefully. First, any future offer of free HIVST kits within a health services context will be a direct offer rather than the possibility (determined by randomisation) of receiving a test and agreeing to complete regular follow-up questionnaires. This raises concerns about generalisability, particularly if some participants joined the trial for altruistic motives, and may affect recruitment to the trial, as a free HIV-test is not guaranteed. Secondly, results will need to be interpreted in the context of the current “standard of care”. When the trial was initially being designed HIVST was illegal in the UK, whereas the BioSURE kits were commercially available when SELPHI launched in February 2017. The study objectives therefore needed to be defined carefully i.e. the effectiveness of offering *free* tests rather than offering tests per se. A related issue is the impact of other self-sampling and self-testing initiatives from other organisations in the UK.

Another moot design point was whether sending testing reminders should be an intrinsic part of the intervention or whether this should be considered as a separate intervention. We considered a factorial design but eventually decided that the HIVST kits and testing reminders should constitute a single “package”, reflecting the likely promotion of HIVST kits during implementation and to maximise the chance of demonstrating an effect. A residual concern is that the regular 3-monthly questionnaires will act as a reminder for participants in the nRT group to seek an HIV test and reduce the difference between the groups. However, we expect that a large proportion of participants in this group will opt out of receiving emails or ignore the follow-up surveys. This highlights the importance of determining the study primary endpoint from an independent national surveillance database to mitigate potential selective survey completion bias. A similar linkage to the PHE national dataset has been conducted to identify additional infections in the long-term follow-up of participants in the PROUD trial of HIV pre-exposure prophylaxis [28]. Reassuringly, of the 32 participants who were diagnosed in one of the PROUD clinics, only 1 (3%) was not identified in the national surveillance database [29]. The sample size calculation for SELPHI factored in a linkage failure rate of 10%. Another consideration is the delay in the centralisation and reconciliation of reports of HIV diagnoses from clinics and laboratories across the country, and a finalised dataset for a given calendar year is usually not available until June of the following year.

SELPHI is expected to complete in 2020 although the results of Randomisation A should be available earlier than this. As well as the main randomised comparisons, we are undertaking qualitative sub-studies of SELPHI participants, process evaluation and developing cost-effectiveness models which will be informed by the results of the trial.

With expanded prevention and treatment options available for people living with HIV, the need for testing and diagnosis is more important than ever. The reality of shrinking NHS resources and radical cuts and changes to sexual health service provision will require increased innovation with cost saving services such as e-health and postal services. This shift will increase the relevance of results from this trial.

## Abbreviations

ART: Antiretroviral Treatment
CAB: Community Advisory Board
CE Mark: “Conformité Européene” which literally means “European Conformity” Mark
GUM: Genitourinary Medicine
HIVST: HIV self-test
PLWH: People Living with HIV
PPI: Patient and public involvement
RCT: Randomised controlled trial
REC: Research Ethics Committee
SELPHI: An HIV Self-testing Public Health Intervention
STI: Sexually transmitted infection
UK: United Kingdom
UN: United Nations
